# Use of Herring Bait to Farm Lobsters in the Gulf of Maine

**DOI:** 10.1371/journal.pone.0010188

**Published:** 2010-04-15

**Authors:** Jonathan H. Grabowski, Erika J. Clesceri, Adam J. Baukus, Julien Gaudette, Matthew Weber, Philip O. Yund

**Affiliations:** 1 Gulf of Maine Research Institute, Portland, Maine, United States of America; 2 U.S. Agency for International Development, Washington, D. C., United States of America; 3 University of Southern Maine, Portland, Maine, United States of America; 4 Monhegan Island, Maine, United States of America; 5 University of Maine, Darling Marine Center, Walpole, Maine, United States of America; University of North Carolina at Chapel Hill, United States of America

## Abstract

**Background:**

Ecologists, fisheries scientists, and coastal managers have all called for an ecosystem approach to fisheries management, yet many species such as the American lobster (*Homarus americanus*) are still largely managed individually. One hypothesis that has yet to be tested suggests that human augmentation of lobster diets via the use of Atlantic herring (*Clupea harengus*) as bait may contribute to recent increases in lobster landings. Currently 70% of Atlantic herring landings in the Gulf of Maine are used as bait to catch lobsters in traps throughout coastal New England.

**Methodology/Principal Findings:**

We examined the effects of this herring bait on the diet composition and growth rate of lobsters at heavily baited vs. seasonally closed (i.e., bait free) sites in coastal Maine. Our results suggest that human use of herring bait may be subsidizing juvenile lobster diets, thereby enhancing lobster growth and the overall economic value and yield of one of the most valuable fisheries in the U.S.

**Conclusions/Significance:**

Our study illustrates that shifting to an ecosystem approach to fisheries management should require consideration of cross-fishery interactions.

## Introduction

With the acknowledged failure of many single-species management strategies, scientists and managers are increasingly adopting a multi-species approach to understanding fisheries ecosystems [1]–[6]. There has been an increasing awareness that the population dynamics of different species of commercially harvested fish are likely to be linked, and these possible connections have led to repeated calls for ecosystem-based management [7]. Many of these connections occur because of direct ecological interactions between species in nature. However, fishing practices can also create functional linkages between species, even if little interaction exists in nature. For example, any species fished as bait for another industry is essentially involved in a predator-prey interaction (albeit mediated by man) even if such predation events would rarely occur in nature. Changes in the abundance of either species can thus affect the abundance of the other, with corresponding consequences for fishing pressure. One such pair of harvested species is the American lobster (*Homarus americanus*) and Atlantic herring (*Clupea harengus*), which is used as bait in lobster traps throughout northeastern U.S.

The New England fishing communities have long suggested that they are effectively farming lobsters. Lobster landings in the Gulf of Maine have achieved levels that are higher than historically thought to be sustainable, with U.S. landings steadily increasing since 1989 to ∼37,500 MT from 2001 to 2008 [8]. Landings in Maine's state waters have surged to 21–34,000 MT per year since 1997, which is more than double the 40-year annual average of ∼9,000 MT from 1950 to 1990 [9]. The value of lobster landings in Maine averaged just over $250 million during 2001–2008, and accounted for almost two-thirds of the state's total fishery value. Identifying the mechanism responsible for increases in lobster populations over the past two decades is particularly important because lobsters are major predators in nearshore waters of the Gulf of Maine and currently constitute one of the most valuable fisheries in the United States [10].

Herring bait, which could subsidize lobster populations by increasing individual growth, survivorship, and fecundity, enters the lobster diet in at least three ways: a) consumption by undersize lobsters that escape or are released from traps, b) consumption by adults that subsequently escape traps (video monitoring of lobster traps indicated that over 90% of juvenile and adult lobsters caught in traps escape) [11], and c) consumption of discarded bait by both juveniles and adults. Trap densities have increased almost four-fold over the last two decades, indicating that bait use has also increased dramatically in coastal Maine [12]. Approximately 100,000 MT of herring are landed in New England each year (106,000 MT in 2000), and about 70% of the herring landings (70–75,000 MT) go back into coastal waters as lobster bait. Atlantic herring accounted for the vast majority of total bait used in recent years [12].

Other hypotheses have been advanced to explain the increase in lobster landings. The predator reduction hypothesis maintains that lobster populations have surged because the overexploitation and subsequent collapse of natural predators like the Atlantic cod has released juvenile lobsters from substantial predation pressure [5]. The importance of sheltered habitats to juvenile lobsters supports this hypothesis [13], [14], and catches of crustacean prey in this region are inversely correlated with that of cod [15]. Ecologists have also demonstrated empirically that crustacean mortality rates are higher on offshore ledges where groundfish are still abundant, whereas mortality rates were much lower in coastal regions of the Gulf of Maine where groundfish are less abundant and smaller [16], [17].

Trends in lobster and groundfish landings over the past century also suggest that groundfish may exert top-down control on lobsters. Cod fishing in coastal waters intensified in the 1930's with the advent of refrigeration and coastal cod stocks in the Gulf of Maine were considered depleted by 1950 [17]. In contrast, lobsters, which experienced a precipitous decline in landings between 1920 and 1950, began increasing in abundance by the end of the 1940's. In the 1990's, lobster landings began increasing again, which coincided with the implementation of additional groundfish fishery reductions throughout the Gulf of Maine. These trends collectively suggest that the reduction in predators may at least partially explain why lobster abundance is currently high, and should be considered in concert with other possible explanations.

Other important factors that may have influenced lobster landings include water temperature, changes in lobster management, and recruitment. However, changes in physical factors such as water temperature do not adequately explain recent trends in lobster population dynamics throughout southern New England, Maine, and the Maritime Provinces of Canada [12], [18]. No major regulatory changes occurred during this period [19]. Strong correlations between new recruits and both juvenile lobster abundance and commercial landings along the coast of Maine suggest that recruitment at least partially limits lobster populations in the Gulf of Maine [10], [20]. Disentangling the multiple factors that might influence lobster recruitment and be responsible for recent population increases remains a major challenge for fisheries ecologists and managers.

In this study, we evaluated how much herring bait contributes to the production of lobster biomass by quantifying the diet composition (via nitrogen stable isotope analyses [SIR's] and stomach content analysis) and growth (with a mark-recapture experiment) of sublegal (66–83 mm carapace length [CL]) juvenile lobsters in areas with vs. without bait in mid-coast Maine in 2002 through 2004. Monhegan Island (MI) served as a bait-free area because its lobster fishery closes seasonally from the end of May until early December, when lobsters are actively growing [21]. Subtidal (10–15 m depth) sites around MI were compared to proximal sites around the Georges Islands (GI), where fishing remains intense from the spring through the fall. MI is subject to a unique management plan, and hence is the only seasonally bait-free area available for study.

Nitrogen SIR's (δ^15^N) measure an organism's trophic position and prey assimilation (as opposed to simple intake) [22]. Analysis of nitrogen SIR's of the three most common natural prey of lobsters (i.e., crabs, sea urchins, and molluscs) [23] at our study sites determined that δ^15^N values for herring were 1.2–5.6 ‰ higher than for natural prey as a consequence of isotopic trophic enrichment (herring: 11.7±0.1 δ^15^N [mean ±1 standard error]; Cancer crabs: 10.5±0.1 δ^15^N; urchins: 6.1±0.2 δ^15^N; and mussels: 7.7±0.2 δ^15^N). This trophic fractionation (δ^15^N difference between consumer and food) suggests that herring are about 0.5 to 1.5 trophic levels above typical lobster prey [24]. This premise is also supported by information on the feeding biology of these species. Sea urchins and mussels are primarily herbivores, while crabs are primary carnivores that largely feed on urchins, molluscs, and other herbivores. By contrast, herring feed mainly on herbivorous (as juveniles) and carnivorous (as adults) copepods, thus shifting from primary to secondary carnivores during ontogeny. Consequently, the nitrogen SIR of lobster tissue serves as a chemical tracer that can be used to indicate whether herring or other higher trophic level prey is a prevalent component in the diet of lobsters. Although C SIR's are often used to identify dietary sources, C SIR values did not differ for lobsters reared for four months on diets of herring and mussels (See Methods). Use of the N SIR tracer to infer diet trends coupled with growth information from lobster mark-recapture experiments at baited vs. seasonally bait free sites in coastal Maine permitted us to evaluate whether herring bait augments lobster stocks in the Gulf of Maine.

## Results

### Herring dietary contribution

**Figure 1:**
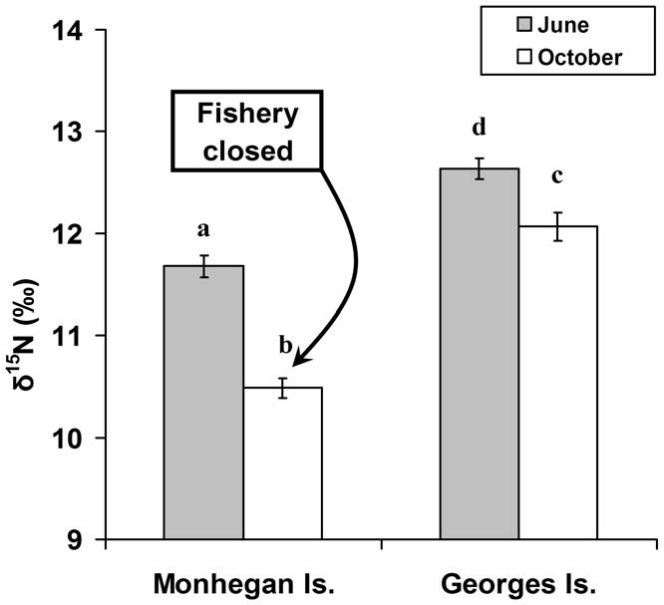
The effects of herring bait on the diet of lobsters in coastal Maine. Nitrogen stable isotope ratios [SIR's] for sublegal (66–83 CL) lobsters in the summer and fall from closed and open sites in mid-coast Maine (n = 10; error bars  = +1 SE). ANOVA revealed a significant interaction between site and season. Nitrogen SIR's of Monhegan lobsters were significantly lower in October than in June, but did not differ at the baited (GI) site. Higher δ^15^N values for lobsters indicate greater proportions of higher trophic levels such as herring in their diet.

Lobsters were collected via SCUBA and dissected at both seasonally bait-free (MI) and baited (GI) sites in early June directly after the Monhegan Island fishery closed (May 31) and then again in October after several months of closure. A two-way ANOVA revealed a significant interaction between site and season on nitrogen SIR values (site x season interaction: F_1,100_ = 6.6, p = 0.01). Year effects were not significant and consequently were removed from the final model. Nitrogen SIR values were significantly lower for MI lobsters from October (after 5 months without bait) than for MI lobsters from June shortly after fishing stopped (Figure 1; Ryan's Q test: p<0.05). In each season, SIR values of GI lobsters were significantly greater than those from MI (Ryan's Q test: p<0.05). However, the magnitude of this effect was much more pronounced in the fall than in June (Figure 1) after lobster fishing had been closed at MI. Because nitrogen SIR's for the natural prey of lobsters are substantially lower than that of herring, these results suggested that lobster consumption of herring was common at our sites, except when lobster fishing was prevented at MI. δ^13^C values of lobsters, which should reflect differences in the source of carbon fixed via photosynthesis, did not differ between the sites.

**Figure 2:**
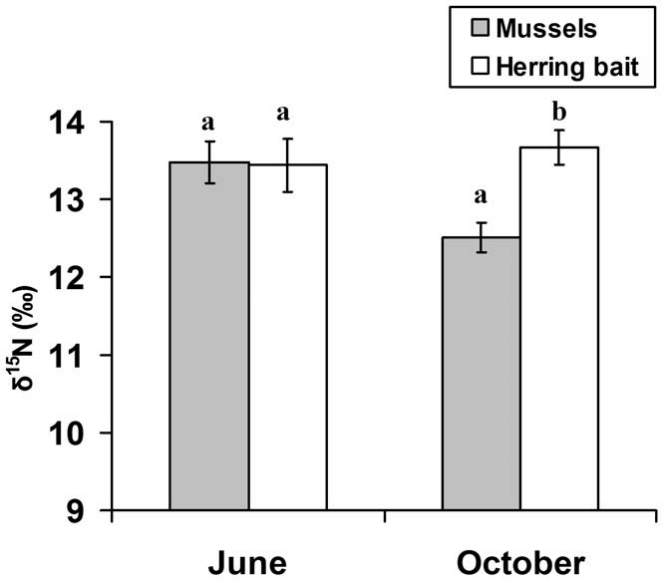
The effects of herring bait on lobster N SIR values. N SIR values of lobsters reared on herring bait (n = 5) vs. mussels (n = 6) in the laboratory at the Darling Marine Center (error bars  = +1 SE). N SIR values were similar at the start (June) of the experiment, but lobsters that consumed mussels were significantly lower at the end (October) of the experiment. Letters denote the results of Ryan's Q post hoc tests–bars with different letters were significantly different at p<0.05.

Lobsters were raised in the lab in the summer and fall of 2004, and were provided diets of herring vs. natural prey to examine if nitrogen SIR's are influenced by diet. This experiment determined that N SIR's indicate differences between fish and natural prey diets in lobsters; there was a significant interaction between time and diet in a repeated measures ANOVA (diet x time: F_1,9_ = 14.5, p = 0.004). There was no difference between the N SIR of lobsters at the beginning of the experiment, but the δN^15^ of lobsters that consumed natural prey was significantly lower than those that ate herring bait (Ryan's Q tests: p<0.05; Figure 2). The magnitude of this decline was fairly comparable to the decline that occurred in lobster SIR's at MI in the fall five months after the fishery was closed. By contrast, there was no effect of either diet on δC^13^ values of lobsters in the lab experiment (diet x time: F_1,9_ = 1.9, p = 0.20; diet: F_1,9_ = 2.3, p = 0.16).

**Figure 3:**
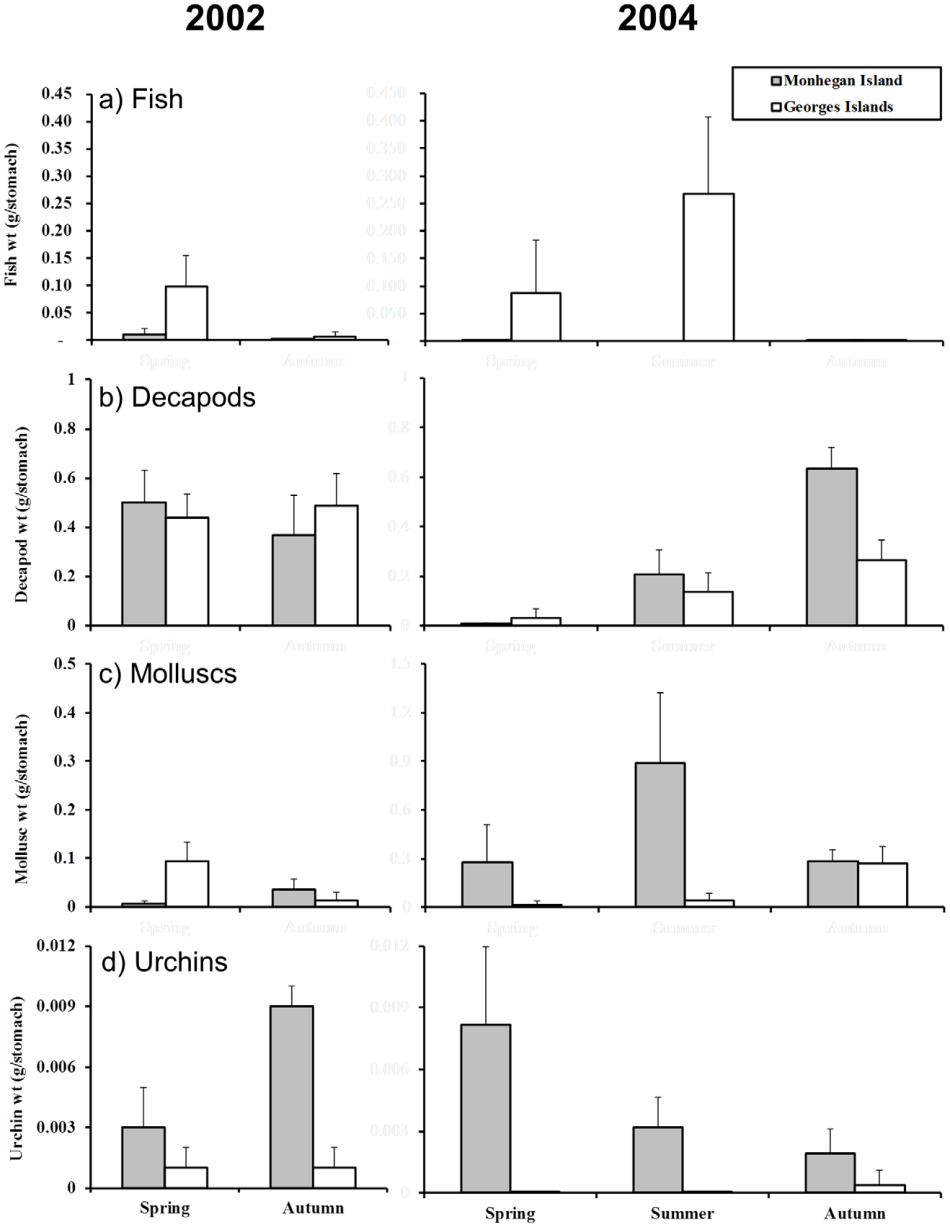
The diet composition of lobsters from Monhegan and Georges Islands. The effects of season (spring and autumn in 2002; spring, summer and autumn in 2004) and site (Monhegan Island vs. the Georges Islands) on a) fish, b) decapod, c) mollusc, and d) urchin biomass found in stomach contents of lobsters (n = 20 in 2002; n = 8–10 in 2004; error bars  = +1 SE).

Examination of stomach contents of lobsters from MI and GI revealed that the primary difference between these sites was the increased prevalence and amount of fish biomass (i.e., mostly bones) in the diet of lobsters at GI in the summer and fall after the Monhegan lobster fishery closes. Fish biomass was found in 60% of the stomachs of lobsters collected at GI vs. 10% of those from MI. Lobsters from MI contained only trace amounts of fish biomass (0.003 g), whereas those from GI contained over an order of magnitude more fish biomass (0.086 g). In 2002, both season and site significantly affected consumption of fish biomass by lobsters (season: F_1,76_ = 6.1, p = 0.02; site: F_1,76_ = 4.4, p = 0.04), but the interaction between the two was not significant (season x site: F_1,76_ = 0.04, p = 0.85; Figure 3). Lobsters from GI consumed 9.5X as much fish biomass as those from MI, and lobsters in general consumed 16.6X as much in the spring than in the fall. Lobsters from GI again in 2004 consumed significantly more fish biomass than those from MI (F_1,48_ = 4.2; p = 0.046). There was a marginally significant interaction between season and site (F_2,48_ = 3.0; p = 0.06). In particular, lobsters at GI consumed 126.0X more fish biomass in the spring and several orders of magnitude more in the summer (Ryan's Q tests, p<0.05), but fish consumption by lobsters did not differ between sites in the fall when bait consumption was negligible (Ryan's Q test, p>0.05).

Natural prey did not substantially differ in the diet of lobsters from GI vs. MI. In both years, decapods and molluscs (mostly shell fragments) were the 2 most common components of the diet of lobsters, and accounted for ∼60% of the natural prey biomass in the stomach contents of lobsters examined in this study. Most of the remaining biomass consisted of unidentified tissue and shell fragments. In 2002, neither decapod nor mollusc biomass differed between sites or seasons (p>0.05 for both main effects and the interaction between season and site for both analyses). In 2004, only season significantly influenced the amount of decapod biomass in the stomach of lobsters (F_2,48_ = 8.4; p = 0.0008). Lobsters in general consumed more decapod biomass in the summer and fall than in the spring (Ryan's Q tests; p<0.05). For molluscs in 2004, only the site effect was significant (F_1,48_ = 9.0, p = 0.004), and lobsters from MI consumed 4.4X more molluscs than those from GI. Site was the only significant effect for the analyses of echinoderm biomass consumed. In 2002, lobsters from MI consumed marginally more echinoderm biomass (F_1,76_ = 3.4, p = 0.07), whereas this effect was significant in 2004 (F_1,48_ = 14.0; p = 0.0005). Lobsters from MI consumed 6.0 (2002) to 31.3 (2004) X more urchin biomass than those from GI. In general, echinoderms occurred in <50% of lobsters, and only trace amounts of urchin tests and spines were found when they were present.

In order to determine the proportion of lobster production derived from herring bait, we first estimated a fractionation coefficient (measure of trophic transfer enrichment) for nitrogen isotopes because animals tend to disproportionately retain heavier isotopes and have heavier nitrogen isotope values than their dietary members. We calculated the fractionation coefficient by subtracting the average value of the natural prey from Monhegan lobsters that were sampled in the fall. Monhegan lobsters sampled in the fall have not had access to herring bait for several months and should have tissue signatures indicative of a diet of natural prey. Suring and Wing [25] estimated muscle tissue turnover in red rock lobsters (*Jasus edwardsii*) at 147 days. Because values for natural prey vary (i.e., crab values tended to be higher than urchins, molluscs, etc.) and previous dietary studies have suggested that variability exists in the diet composition of lobsters, we calculated fractionation coefficients for a range of lobster diets. Using dietary information from this study and previous investigations [26]–[30], we estimated that crabs typically account for at least 50% of the diet of lobsters and that crabs, molluscs (i.e., mussels, clams and small scallops), and urchins are the three most important components of the diet.

**Figure 4:**
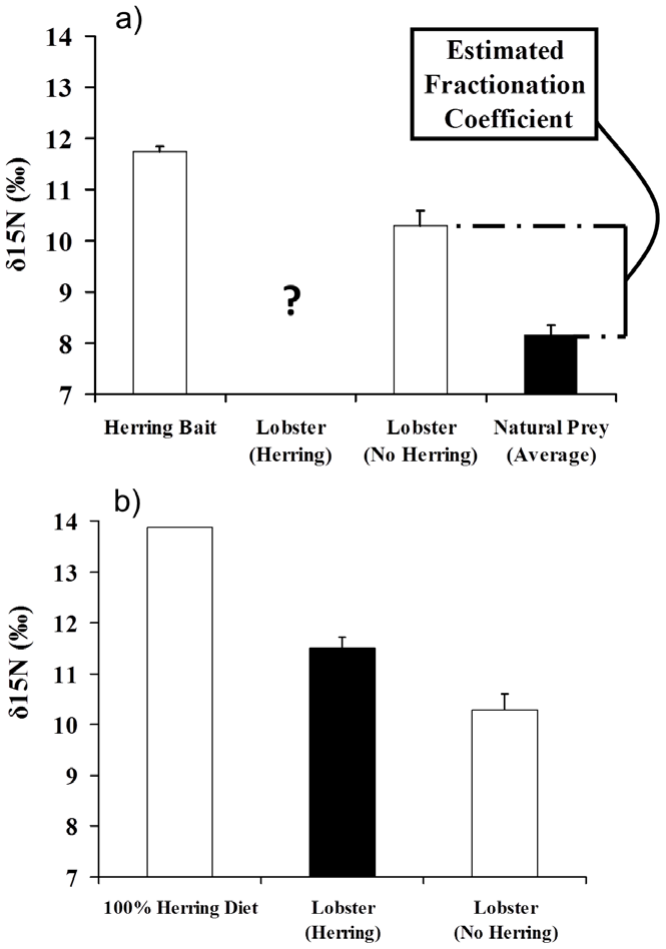
The effects of herring bait on lobster growth at the Georges Islands. Lobster growth rates at MI (seasonally closed) and GI (open) sites in 2002 (n = 44) and 2004 (n = 92). Lobsters at GI consistently outgrew those at MI even though lobster growth rates at both sites were lower in 2004 than in 2002 (error bars  = +1 SE).

Because crabs tend to account for a larger proportion of the diet of larger lobsters and urchin populations have been widely reduced at our study sites from harvesting efforts, crabs probably account for a greater proportion of the diet of larger lobsters at MI. Fractionation coefficients were calculated for the following two diets: (1) 50% crab, 25% molluscs, and 25% urchins; and (2) 75% crabs, 12.5% molluscs, and 12.5% urchins (see Figure 4 for the derivation of the fractionation coefficient using the 50% diet). Fractionation coefficients varied from 2.4‰ (50% crab diet) to 1.5‰ (75% crab diet). The fractionation coefficient was then added to the value of herring to estimate the value of a lobster diet that is comprised solely of herring. We then used this estimate (100% herring diet) and the isotope values for MI lobsters sampled in October (100% natural prey diet) to determine the importance of herring in the diet of lobsters in mid-coast Maine. In particular, we compared these isotope values to MI lobsters from June, which recently had consumed herring bait, and calculated the relative proportion of lobster tissue derived by herring bait. Utilizing this method for each natural prey diet discussed above, we calculated that herring bait is responsible for deriving 33.3–44.6% of the tissue production of lobsters at GI.

### Herring effects on lobster growth

**Figure 5:**
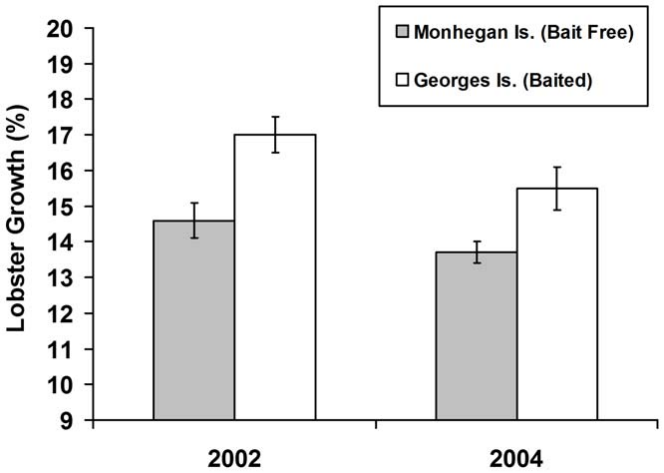
Contribution of herring to the diet of lobsters in mid-coast Maine. (a) Fractionation coefficients were calculated by subtracting the isotopic values of lobster prey from the value of MI lobsters from October that have not consumed herring recently. (b) Isotope values for lobsters that feed only on herring were calculated by adding the fractionation coefficient calculated above for lobsters to herring isotope values. Isotope values of lobsters that recently had access to herring bait (MI June samples) were then compared to the estimated isotopic value of lobsters that feed 100% on herring vs. completely on natural prey (MI October samples) to determine the proportion of herring in the diet of lobsters in mid-coast Maine when fishing is permitted. Error bars denote +1 SE.

Mark-recapture experiments revealed that both year and site (baited vs. bait-free) influenced lobster growth rates independently (i.e., the site x year interaction was not significant: F_1,96_ = 0.2, p = 0.62; Figure 5). Tagged lobsters recaptured from GI (baited) outgrew those from MI (seasonally bait-free) by 14.8% (treatment: F_1,96_ = 11.7, p = 0.0009). This effect was consistent across years even though lobster growth rates in general were greater in 2002 than in 2004 (Figure 5; year: F_1,96_ = 3.9, p = 0.05), presumably because water temperatures in the summer and fall were colder in 2004 (Ocean water temperature data for the summer and fall of 2002 and 2004 available for mid-coast Maine region at www.gomoos.org). In spite of this annual difference in water temperatures, the pattern of greater growth at the baited site was consistent across both sampling years. Moreover, lobsters from GI in 2004 outgrew those from MI in 2002 by ∼10% even though the water temperature at MI in 2002 was warmer than the mean temperature recorded at the buoy nearest to the GI site in 2004 by 0.5–1.2°C at 2–20 m depth. These results collectively suggest that temperature alone can not explain why the growth rates of tagged lobsters from MI and GI differed.

Recapture rates of lobsters that molted were substantially higher at MI (2.4%) than at GI (0.9%). This difference is likely due to reduced reporting of recaptured lobster from GI rather than differences in emigration rates, though we are unable rule out this possibility. Lobsters from MI were recaptured and reported by the ∼15 fishers that were permitted to fish in this region when the fishery was open in 2002–2005. A couple of these lobster fishers also participated in tagging lobsters for this study, and every member of this lobster fishing group was very aware of the study. While the 2 project participants that fish around GI were extremely supportive, they represented a much smaller proportion of the entire fishery at that site. In comparison to MI, several hundred lobster fishers fish traps in the general vicinity of the Georges Islands in mid-coast Maine. These lobster fishers belong to several different coastal Maine communities, each with their own lobster fishing territory. Furthermore, they sell lobsters to dozens of different dealers along the middle of the coast, some of whom decided not to participate in this study.

### Augmented value derived from herring bait subsidy

Using our growth estimates derived from the lobster tagging efforts at MI and GI, we estimated that 14.1% of lobster biomass in 2002 and 11.1% in 2004 was a result of the herring bait subsidy. Extrapolating to landings in the state of Maine, our results suggest that of the 28,860 MT of lobster landings valued at $211.0 million in dockside value in 2002, up to an estimated 4,074 MT worth $29.8 million were derived from herring bait use. In 2004, Maine lobster landings of 32,278 MT were worth $286.7 million, with herring bait predicted to augment landings by an estimated 3,748 MT worth $33.3 million. Restricting these estimates to mid-coast Maine (i.e., from Sagadahoc County to Lincoln County) where sampling efforts were conducted still resulted in an estimated $20.8 million (2,539 MT) in 2002 and $25.5 million (2,512 MT) in 2004 of additional value to the lobster fishery from use of herring bait. As an alternative approach, we multiplied the annual herring landings of 103,396 MT by 70% to estimate that the annual amount of herring biomass used by the lobster fishery between 2000 and 2004 was 72,377 MT and multiplied this amount by an estimated trophic efficiency of 10% to calculate total potential augmented biomass of lobsters at 7,238 MT. Potential augmented biomass exceeded our estimates derived from empirical data by 85.4%, probably because our implicit assumption that 100% of herring bait was consumed by lobsters was violated and since some of this herring was used by the portion of the U.S. lobster fishery that is south of Maine.

### Examination of possible confounding factors that may have influenced our results

**Figure 6:**
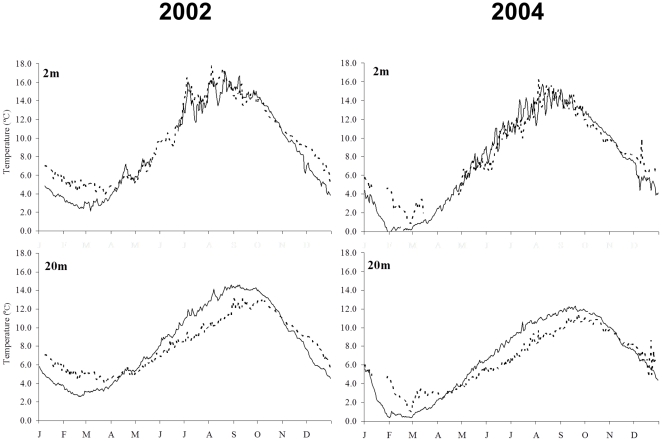
Bottom water temperatures in mid-coast Maine. Mean daily temperature data collected at MI and from Penobscot Bay (proxy for GI) from 2002 and 2004. Temperature was collected at depths of 2 and 20 m by the Gulf of Maine Ocean Observing System and are available at www.gomoos.org.

To determine if factors other than herring bait may have influenced lobster growth rates at MI and GI, we examined bottom water temperature, habitat types, lobster densities, and prey availability (Figure 6). In 2002, the daily bottom water temperature at 2 m was significantly warmer at MI in the winter by 2.1°C (p<0.01), did not differ in spring (p>0.01), and was slightly warmer at MI by 0.5°C in summer and 0.7°C in fall (p<0.01 for both tests). Temperature fluctuated among sites more substantially at 20 m. MI was warmer in the winter and fall by 1.9°C and 0.4°C, respectively, but was cooler in the spring by 0.4°C and the summer by 2.4°C (p<0.01 for all tests). Although bottom water temperatures were generally cooler in 2004 than in 2002, seasonal trends and differences between MI and western Penobscot Bay in 2004 were consistent with those in 2002. At 2 m, bottom temperature was warmer at MI in winter by 2.1°C (p<0.05, cooler in the spring by 0.6°C (p<0.05), and did not differ in the summer and fall (p>0.05 for both tests). At 20 m, bottom water temperature again was warmer at MI in the winter by 1.7°C, but was cooler in the spring, summer and fall by 0.6, 2.0, and 0.2°C, respectively (p<0.01 for all tests). In general, temperatures variations were slight except in the winter when MI was generally warmer and at 20 m in the summer when MI was slightly cooler than in western Penobscot Bay. Given that the Georges Islands are located further offshore than the buoy in Penobscot Bay and closer to Monhegan Island, the slight water temperature differences observed at these buoys are likely an overestimate of the actual differences in water temperature between MI and GI.

We examined whether lobster densities differed between sites because if densities were lower at the baited site, then these lobsters may grow faster than those at the closed site because of reduced competition for food. We also quantified seasonal patterns in lobster densities to determine if the length of time that the fishery is closed at MI influences lobster density patterns between our sites. In 2002, closure status (F_1,60_ = 0.2, p = 0.66; open: 0.6±0.1; closed: 0.6±0.1), season (F_1,60_ = 1.5, p = 0.23), or their interaction (F_1,60_ = 0.3, p = 0.61) did not affect the density of lobsters recorded in diver surveys. Again in 2004, there was no effect of site on lobster density (F_1,234_ = 2.3, p = 0.13). However, there was a significant effect of season on lobster density (F_2,234_ = 8.2, p = 0.0004), with densities higher in the summer (1.0±0.1 SE) and fall (1.1±0.1 SE) than in the spring (0.6±0.1 SE). The interaction between site and season was marginally significant (F_2,234_ = 2.3, p = 0.10). Mean densities did not differ between sites in the spring or summer, but were higher at the closed site in the fall (open: 0.9±0.1 SE; closed: 1.4±0.1). Lobster densities only differed between MI and GI in the fall of 2004, which is well after the seasonal increase in molting frequency that typically occurs during the summer.

**Figure 7:**
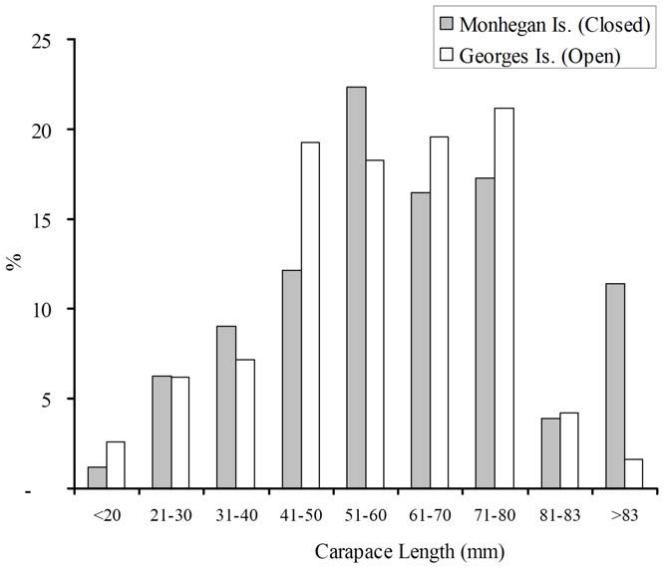
Size-frequency distributions of lobsters from Monhegan and Georges Islands. The Carapace Length of lobsters captured in 1 m^2^ quadrat diver surveys conducted at MI and GI in 2004 was measured in order to compare the size-frequency distributions of lobsters between sites.

The overall size distributions of lobsters collected in 2004 were consistent between sites (MI: n = 255; GI: n = 307, Kolmogorov-Smirnov test Chi square = 5.8; p = 0.11). Legal-size lobsters (>83 mm CL) accounted for a larger percentage of lobsters at MI, but the next three largest size classes were more common at GI (Figure 7). The mean size of lobsters varied together with season and site (significant season x site interaction: F_2,556_ = 3.1; p = 0.045). The mean size of lobsters did not differ in the spring and the summer (spring: MI = 62.8±1.8; GI = 60.9±1.2; summer: MI = 62.4±2.1; GI = 59.6±1.9; Ryan's Q tests p>0.05), whereas lobsters were larger at MI in the fall (MI = 56.9±2.8; GI = 45.6±2.0; Ryan's Q test p<0.05). These results suggest that the size distributions and densities of lobsters were consistent between sites, and thus likely cannot explain why lobster growth rates at MI and GI differed.

Habitat characteristics did not differ substantially between sites. In particular, the proportion of cobble/boulder and algae observed in quadrat samples did not differ between MI and GI (cobble/boulder: Z = −0.6, p = 0.56; algae: Z = −1.5, p = 0.15). Cobble/boulder typically covered at least 50% of the quadrat at both sites (mean rank at MI = 2.8; GI = 2.7), whereas algae cover was slightly lower than cobble/boulder (MI = 2.3; GI = 1.9). The proportion of quadrats covered by shell material was significantly greater at MI than at GI, but shell cover was low at both sites: MI = 0.7; GI = 0.5 (Z = −2.2, p = 0.03). Finally, shelter availability did not differ between sites: MI = 1.6; GI = 1.6 (Z = −0.3, p = 0.80).

Prey densities at MI and GI suggested that prey availability is comparable, although some prey groups did differ between sites. Gastropod densities did not differ between sites (F_1,66_ = 1.6, p = 0.22), but did vary with season (F_2,66_ = 4.5, p = 0.01). Gastropods were most common in the fall (2.2±1.0 per m^2^), intermediate in the spring (0.8±0.5 per m^2^), and absent in the summer. Bivalve densities significantly differed between sites (F_1,66_ = 151.2, p<0.0001), and were more abundant at GI than at MI (MI = 17.0±3.3, GI = 198.6±14.2 per m^2^). However, the vast majority of these bivalves were large (i.e., >50 mm shell length) horse mussels that existed in hummocks and thus were probably not available for consumption by lobsters. Examination of the density of bivalves other than adult horse mussels suggested little difference between MI and GI (F_1,66_ = 1.1, p = 0.29). Urchin densities did not differ between sites (F_1,66_ = 0.7 p = 0.39), but there was a trend (F_2,66_ = 2.9, p = 0.06) of more urchins in the fall (4.5±2.0 per m^2^) then the spring (1.3±0.7 per m^2^) or summer (0.5±0.4 per m^2^). Finally, crab densities varied with both site (F_1,66_ = 8.4, p = 0.004) and season (F_2,66_ = 4.8, p = 0.009). Crab densities were higher at MI (0.8±0.1 per m^2^) then at GI (0.5±0.1 per m^2^). Similar to gastropods and urchins, crab densities were highest in the fall (0.8±0.1 per m^2^), intermediate in the spring (0.6±0.1 per m^2^), and lowest in the summer (0.4±0.1 per m^2^). The interaction between site and season was not significant for any of the different prey categories (p>0.10 for all site * season interactions).

## Discussion

In general, biomass production can be augmented by two potentially independent mechanisms: Either (1) by a population increase or (2) by increased growth rates. Our study investigated whether this second mechanism explains a portion of the increased lobster landings in Maine. We found that lobsters at our baited site (GI) outgrew those at the closed site by 15%. Results from nitrogen SIR and stomach content analyses indicate that fish such as herring is an integral component of the diet of sublegal lobsters in the Gulf of Maine.

These findings are based on a single pair of sites because we were unable to adequately replicate our control (i.e., bait-free) site in mid-coast Maine. Currently, the waters surrounding Monhegan Island constitute the only seasonal closure in coastal Maine. This limitation of our experimental design has important implications for the interpretation of our analyses. Hurlbert [31] warned against the use of inferential statistics when treatments are not replicated, even if the samples may be. Oksanen [32] countered this argument by pointing out that ecologists must choose between sacrificing the appropriate spatial and temporal scale at which to conduct an experiment in order to achieve adequate replication vs. using the appropriate scale without adequate replication. Given that lobsters are extremely mobile, we chose conducting a large-scale, unreplicated experiment over using mesocosms or cages that confine lobsters to a small amount of area and potentially bias their behavior. Although this latter approach may prove to be informative, we question whether it would reveal large scale processes that seem to be inherent in our system. To the degree possible, we have attempted to rule out possible confounding factors such as bottom temperature, lobster density, natural prey availability, and habitat types that could explain why we found differences in the diet and growth of lobsters between our sites (see Materials and Methods and Results sections entitled ‘Examination of possible confounding factors that may have influenced lobster growth). We have also attempted to avoid over-extrapolating these results given the limitations of inadequate replication [31], [33].

Additional approaches have been suggested when scientists are incapable of achieving adequate replication. For example, one possible approach would be to compare a single treatment with replicated controls [32]. While we are unable to create a replicate bait-free area, this study would benefit from future investigations of lobster growth rates at other heavily baited sites in mid-coast Maine. Another approach involves the use of meta-analysis [32], [33], which may be appropriate to address the broader question of whether bait is augmenting fisheries globally. The one other published study of which we are aware of used a mass-balance equation and estimated that bait accounted for up to 13% of the diet of western rock lobsters in Western Australia [34]. Although the sample size is too small and inhibits the use of meta-analytical approaches, the concordance in results between this and our study suggest a more general mechanism.

While the use of herring bait in lobster traps is the most plausible explanation for the increase in fish in the diet of lobsters in GI, we cannot rule out other possibilities. Lobsters could be consuming herring that are available on the bottom after natural mortality events. Yet the availability of herring from natural mortality events is likely very low, especially when compared to the amount of herring entering the system via traps. The abundance of many forage species including Atlantic herring, alewives, and sardines is greatly reduced in coastal Maine compared with historical levels [35]. The relative absence of fish in the diet of lobsters from MI also suggests that it is not typically an important dietary source in areas when fishing is closed. Although lobsters are scavengers and consume a wide diversity of prey, previous studies have demonstrated that crabs, bivalves, and urchins are the most important natural sources of prey in their diet [23], [26], [36].

Lobsters at GI may also be consuming other species of fish that reach the bottom through natural mortality events or that are discarded from the lobster and other fisheries. Yet populations of many other traditionally important fish predators such as cod, cusk, wolffish, and Atlantic halibut currently are also greatly diminished in coastal Maine [37], so it is unlikely that much natural or anthropogenic biomass is available to lobsters from these sources. The current number of fishers in these fisheries in coastal Maine has diminished to a small percentage of the historical abundance. Lobsters could also be consuming carrion from fish that are still prevalent in coastal Maine such as cunner (*Tautoglabrus adspersus*) and sculpins (*Myoxocephalus* spp.), but the fish bones found in our stomach content analyses resemble herring bones more than either of these other species groups. Finally, while the lobster fishery uses species other than herring as bait, most of these have been filleted and are present as skeletons with minimal residual tissue. Thus they provide a scent to attract lobsters but little available food, unlike when herring is used as bait.

Recently it has been argued that herring bait subsidies have little to no effect on lobster landings because increases in lobster landings in Canada have been commensurate with those in the U.S. over the past three decades even though the Canadian fishery put a ceiling on effort in the 1970s [38]. However, it is currently unclear whether lobsters landed in Canada are food limited and subsequently if landings would have been greater had the Canadian fishery continued to ramp up effort over the past 3 decades. Moreover, it is difficult to compare the ecology of lobsters from these two regions given the differences in the physical (i.e., water temperature, circulation patterns, benthic habitat, etc.) and biological (i.e., food availability, predator regimes, lobster densities, etc.) characteristics of the U.S. and Canadian waters. Furthermore, the amount of herring and other fish introduced as meal for salmon aquaculture has intensified over the past two decades in regions of eastern Canada such as the Bay of Fundy [39], which could serve as a bottom-up stimulus to Canadian lobster fisheries in lieu of trap increases.

A recent investigation attempted to replicate the current study using a site that is fished all year in eastern Maine and a seasonally closed site in the Bay of Fundy, Canada [40]. In this earlier study, growth was not augmented at the baited site. However, bait use is much lower in eastern Maine than it is in central Maine, suggesting that there may be a threshold over which bait augments lobster abundance. The sites used in this previous study were also much further apart to avoid the high abundance of aquaculture sites along the New Brunswick, CA-Maine, U.S. border. Several differences among the sites confounded whether bait is important, including natural prey availability, bottom water temperature, and tidal regime. The study did conclude that lobsters in this region are food limited, so that their growth would likely be influenced by fluctuations in natural prey and other sources such as bait from traps. This recent study also illustrates the difficulty of comparing lobster population dynamics in Canada and the U.S.

We have inferred that herring bait is augmenting lobster populations at GI, and potentially throughout coastal Maine where fishing is intense, whereas natural prey resources are likely limiting lobsters in the absence of bait subsidies at sites such as MI. Increased lobster production as a consequence of herring bait is likely reflected in greater landings because lobsters achieve legal size more rapidly and are potentially larger when harvested. Adult lobster mortality is most common either during or directly after moulting, so that requiring fewer moults to achieve legal size should increase the likelihood that a lobster survives until it is large enough to be harvested. Herring bait could also increase moulting frequency, which would increase the likelihood that lobsters that are legal size moult again prior to being landed. The lobster fishery currently accounts for a disproportionately large percentage of the total fishery value in the Gulf of Maine and was the single most valuable fishery in the United States between 1998 and 2004 (data on annual landings and economic values for U.S. fisheries from 1950–2006 available at www.st.nmfs.gov/st1/commercial/landings/annual_landings.html). Our results suggest that herring bait augments lobster landings by ∼$20–25 million annually in mid-coast Maine. This estimate is likely conservative because we only measured growth change as a function of carapace length, which is a linear measurement, but weight gain scales with overall lobster size (a volumetric measurement). This estimate may also be conservative because we are capturing growth augmentation over one moult cycle, whereas lobsters consume bait for 2–4 moult cycles prior to achieving legal size. Although herring bait is also eaten by seals, crabs, fish, sea birds and other benthic fauna, our results suggest that lobsters are significant consumers of bait, with substantial economic consequences. Further economic analyses are needed to examine whether the costs associated with fishing at the current effort level are outweighed by this augmentation in lobster biomass from herring bait use.

Increasing awareness that the population dynamics of different commercially harvested species are potentially linked has garnered support for multi-species or ecosystem-based management strategies [3]–[5]. Our results illustrate that managing multiple fisheries should require consideration of cross-fishery interactions and their resultant effects on food web dynamics, and provide evidence that even apparently wild fisheries may constitute a communal form of aquaculture. By augmenting lobster populations, which are primarily fished within 5–10 km of the coast, herring subsidies are likely having additional indirect effects on interactions between lobsters and their natural prey. The lobster fishery has motivated over the past decade removal of ∼10% of total herring stock biomass and up to 30–40% of that which is available in the Gulf of Maine [35]. Thus an enormous amount of biomass is transferred from the pelagic zone to the benthos. Interactions between these fisheries have ultimately increased the strength of benthic-pelagic coupling in the Gulf of Maine ecosystem.

Because herring are typically caught further offshore (i.e., 25–50 km) in the Gulf of Maine, demand for herring from the lobster fishery also results in a net transport of biomass from offshore to nearshore waters. This decrease in herring biomass further offshore could result in either reduced availability of prey resources for slowly recovering species such as Atlantic cod or removal of predators and competitors of larval cod [41]. In other regions of the North Atlantic, cod condition, growth, and fecundity, which are potential indicators of stock productivity [42], have been linked to pelagic forage fish availability [43], [44]. Thus interactions among these three fisheries could have cascading effects in both recipient (nearshore) and donor (further offshore) ecosystems in the Northwest Atlantic. Herring is a critical component of offshore and more recently inshore food webs, so that further investigation of these species interactions is merited.

## Materials and Methods

### Site selection

The Monhegan Island fishery, which encompasses 4–5 km radius around MI, was the only one that was seasonally closed in coastal Maine at the time of the study. Therefore, it was not possible to replicate a herring bait-free area. To avoid confounding our results by choosing sites that included differences other than fishing intensity and herring bait prevalence, we selected the Georges Islands as our baited site because it is proximal to Monhegan Island (i.e., <10 km apart).

### Herring dietary contribution

Lobsters were collected in June and October of 2002 and 2003 and June, August, and October of 2004 for stomach content and stable isotope analyses (number of samples collected per site in each season: stable isotopes: n = 8–10; stomach contents: n = 20). Collected lobsters were dissected to obtain abdominal muscle tissue samples in the field, which were stored on ice in glass scintillation vials until frozen in the laboratory. A subset of samples (n = 8 in 2002 and 2003 and n = 10 in 2004 per each site and season except for summer 2004) were freeze-dried and analyzed for nitrogen SIR's via an elemental analyzer (Carlo Erba NA 1500) interfaced via continuous flow to an isotope ratio mass spectrometer (Finnigan MAT 252). The effects of season (June and October), site (MI and GI) and year (2002, 2003, and 2004) on nitrogen SIR values were tested using a three-way ANOVA. The effect of year was not significant, so this factor was removed from the model and data were reanalyzed using a two-way ANOVA [45]. For the significant interaction between season and site, we conducted Ryan's Q post hoc pairwise tests because this test controls for the experimentwise type I error rate while providing maximum power [46].

During each sampling event, stomach contents were also removed and preserved in 5% formalin for analysis in the laboratory. Total stomach contents were weighed, and individual items were identified, weighed, and enumerated (where possible). Stomach contents were lumped into four categories: fish, decapod, mollusc, and urchin biomass. In 2002, the effects of season (spring vs. fall) and site (MI vs. GI) on each major prey category were analyzed using MANOVA, which was significant (p<0.05), and then separate two-way ANOVAs (n = 20).

Lobsters that are molting are less likely to consume prey, and thus were excluded from the stomach content analyses in 2004. Lobsters form gastroliths, or calcareous sacs, on the exterior lining of their stomachs to preserve calcium during the molting process. We collected and weighed the gastrolith in order to create a molting index by dividing the weight of the gastrolith by the length of the carapace of the lobster. All stomachs where the gastrolith divided by the total length of the lobster exceeded 0.020 were classified as advanced premolt and excluded from stomach content analyses because a precipitous decline in stomach contents was noted in lobsters in this molt phase. Ten of 64 lobsters were excluded using this criterion. Although this index was not utilized in 2002, it was most useful in 2004 for lobsters collected in the summer when molting frequency is high. In 2004, the effects of season (spring, summer, and fall) and site (MI vs. GI) on lobster consumption of fish, decapod, mollusc and urchin biomass were analyzed again using MANOVA followed by separate two-way ANOVAs. In both years, stomach content data required second- to eighth-root transformations to remove heterogeneity of variances.

Sublegal (66–83 mm) lobsters were raised in the laboratory from June until October of 2004 on diets consisting of herring vs. natural prey to determine if nitrogen SIR's are influenced by diet. Lobsters were maintained in captivity in the flowing sea water system of the Darling Marine Center, University of Maine, which is located in Walpole, Maine. Lobsters were housed in 0.5×0.8×0.3 m (l×w×d) sea tables (n = 7 for each diet) with continuous sea water. Lobsters were fed ∼50 g of herring or natural prey (a mixture of horse mussels *Modiolus modiolus* and blue mussels *Mytilus edulis*) every 2 to 3 days throughout the experiment. Lobster tanks were cleaned once a week to remove any recently settled invertebrates that might confound our results.

Lobster tissue was collected for SIR analysis at the beginning of the lab experiment by carefully removing the posterior left appendage from each lobster, removing the tissue from the exoskeleton, and freezing each tissue sample in a glass scintillation vial. At the completion of the experiment, muscle tissue was dissected from the right posterior appendage and the abdomen (similar to above samples) and frozen in separate scintillation vials in order to determine if SIR values vary for these different tissue types. During the course of the experiment, 3 lobsters expired (1 fed natural prey and 2 herring). Because individual lobsters were sampled several times throughout the study, these data were analyzed using repeated measures ANOVA with diet as the main factor and date as the repeated measure.

### Herring effects on lobster growth

In order to assess the effects of herring bait on lobster growth, we conducted mark-recapture experiments at seasonally closed (MI) and open (GI) sites in 2002 and 2004. Prior to the onset of molting that is typical in late summer in the Gulf of Maine, lobsters were tagged in June and early July in each year with streamer tags (Floy Tag Co.–FTSL-73), which were implanted in the muscle tissue directly between the carapace and abdomen. We tagged a total of ∼3000 lobsters in each year in late spring and early summer prior to the molting season at MI and GI (lobsters were measured, sexed, tagged, and released at closed and open sites). Lobsters were recaptured in October of each year via diver collection and trapping at our experimental sites. We also recovered tagged lobsters through a tag reward system with the commercial lobster fishing industry in mid-coast Maine. We quantified the percent growth of those that molted for seasonally closed vs. open sites. The effect of herring bait presence on lobster growth was analyzed using a two-way ANOVA with site (MI and GI) and year (2002 and 2004) as the two factors.

### Augmented value derived from herring bait subsidy

To determine the value of the herring bait subsidy to the lobster fishery, we first used the difference in lobster growth between GI and MI to calculate to the proportional augmentation in lobster growth at GI from herring bait:
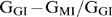
(1)where G_GI_ and G_MI_ were the percent growth of recaptured lobsters at GI and MI, respectively. We then multiplied this estimate of bait augmentation of lobster growth in each project year (2002 and 2004) by the average lobster fishery landings value for Maine during that year to estimate the proportion of landings value attributable to herring bait use. Data on the value of lobster landings in Maine by county from 1964–2006 were obtained at www.maine.gov/dmr/index.htm. Because our study was focused in the central part of coastal Maine, we recalculated this subsidy including only counties in central Maine (Hancock, Knox, Lincoln, Sagadahoc, and Waldo). We then used estimates of herring landings from 2000–2004 [35] to calculate the amount of herring that was used annually as bait. Although it is currently unknown exactly how much of landed herring is used as bait by the lobster industry, fishery scientists have estimated that approximately 70% of herring landings end up being used as bait by the lobster fishery [47], [48]. Finally, we multiplied this estimate of the amount of herring used as bait by an estimated trophic efficiency of 10% [49], [50] to establish the total potential subsidy to the lobster fishery in order to compare it with the estimates derived above.

### Examination of possible confounding factors that may have influenced our results

Because we were unable to replicate this experiment, we examined whether several biotic and abiotic factors other than herring bait availability differed between MI and GI, and consequently could have confounded our results. Water temperature has been shown to influence lobster growth rates [51]. Gulf of Maine Ocean Observing System (GoMOOS) ocean temperature data from this region of the coast was utilized to examine seasonal temperature trends in water at depths of 2 and 20 m near our sites in 2002 and 2004. In particular, ocean water temperature data from western Penobscot Bay (PB), which is slightly (>5–10 km) more inland than our sites and consequently likely results in slightly warmer water than our GI site, was compared with water just south of MI. Paired t-tests were used to examine whether daily temperatures differed seasonally among sites for each depth during both study years.

A second possible confounding factor that could have influenced lobster growth rates is the effect of lobster density. Specifically, if lobster densities were lower at the baited site, then these lobsters may grow faster than those at the closed site because of reduced competition for food. In the summer and fall of 2002 and the spring, summer and fall of 2004, we conducted diver surveys of lobster density using 16 replicate 1-m^2^ quadrats in 2002 and 40 replicate quadrats in 2004 at MI and GI. Quadrats were randomly dropped onto cobble/boulder habitat. All lobsters within each quadrat were collected and counted. The effects of site (MI and GI) and season (2002: spring and fall; 2004: spring, summer, and fall) on the densities of lobsters were analyzed using separate two-way ANOVAs for each year. In addition, the proportion of the quadrat that contained algae, shell, and cobble/boulder habitat was quantified using the following categories (0 = not present; 1 = >0–25% cover; 2 = >25–50% cover; 3 = >50–75% cover; and 4 = >75%–100 cover). The number of quadrants that contained shelter was also counted within each quadrat. Each lobster that was collected in 2004 during quadrat surveys and for diet analysis was measured (carapace length [CL]) in order to compare the size frequency distributions of lobsters at both sites. The overall size distributions of lobsters collected at MI and GI were compared using a Kolmogorov-Smirnov test. Finally, we analyzed the effects of site and season on the mean size of lobsters using a two-way ANOVA.

In addition to choosing locations that were as proximal to each other as possible to avoid differences in water temperature and other environmental variables confounding our results, we attempted to avoid selecting biologically different sampling locations. For instance, we selected locations that contained a mixture of bottom types (rock ledge, cobble/boulder, and sand/mud) at both MI and GI where juvenile lobsters typically aggregate [10]. We were unable to quantify the relative proportion of each habitat type at our sites because bottom habitat maps are currently unavailable for this region of the coast. However, we examined whether the percent cover of algae, shell and cobble/boulder habitat and shelter availability differed between quadrat surveys conducted at MI and GI using Mann-Whitney U tests. These tests are meant to inform whether lobster density surveys were conducted in comparable habitat at both MI and GI.

A fourth possible confounding factor is the possibility that prey densities differed between sites. During lobster surveys in 2002 and 2004, cancer crabs were also collected and enumerated. The effects of season and site on crab densities were analyzed using separate two-way ANOVAs for each year. In the spring, summer, and fall of 2004, we also conducted diver surveys at MI and GI using 0.25 m^2^ quadrats to collect other potential invertebrate prey. All bivalves, urchins, and gastropods were collected and enumerated within each of 12 quadrats conducted at each site per season, and the effect of season and site on each prey category was analyzed using separate two-way ANOVAs. All sampling efforts described above were conducted following the Gulf of Maine Research Institute's guidelines for animal involvement in research.
